# Supplementary material for: Extension of the crRNA enhances Cpf1 gene editing in vitro and in vivo
Source: Nat Commun. 2018 Aug 17;9:3313. doi: 10.1038/s41467-018-05641-3 (PMC6098076; doi:10.1038/s41467-018-05641-3)
Supplement: Supplementary file 1 — Supplementary Information [file 41467_2018_5641_MOESM1_ESM.pdf]

Supplementary Information

Extension of the crRNA enhances Cpf1 gene editing *in vitro* and *in vivo*

Hyo Min Park[1], Hui Liu[1], Joann Wu[1], Anthony Chong[1], Vanessa Mackley[1], Christof Fellmann[3], Anirudh Rao[3], Fuguo Jiang[3], Hunghao Chu[1], Niren Murthy[2]*, Kunwoo Lee[1]*

[1]GenEdit Inc., Berkeley, USA. [2]Department of Bioengineering, University of California, Berkeley, Berkeley, USA. [3]Department of Molecular and Cell Biology, University of California, Berkeley, Berkeley, USA.

*corresponding authors. Email: lee@genedit.com and nmurthy@berkeley.edu

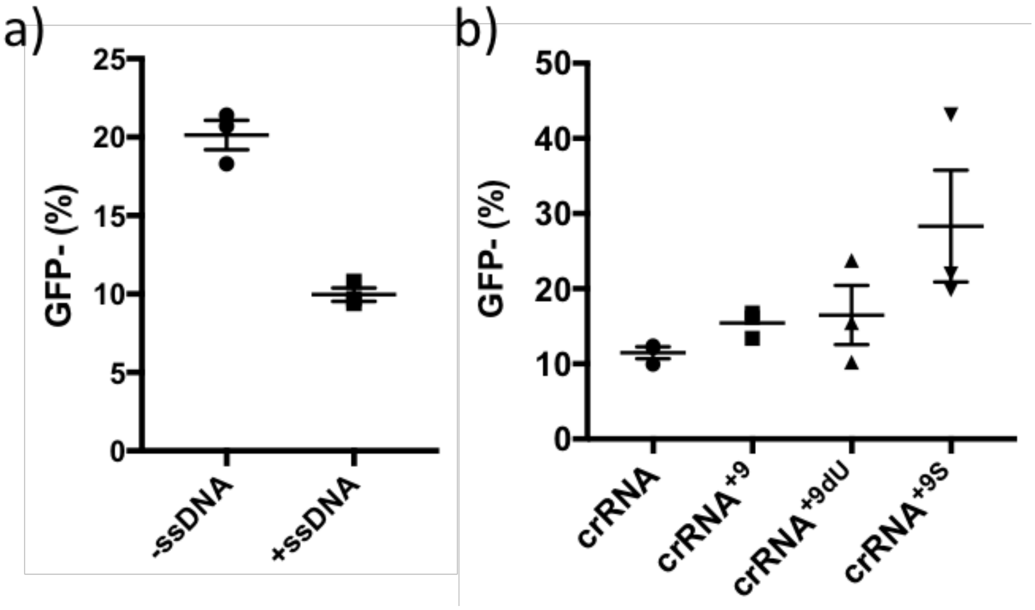

Supplementary Figure 1. Lipofectamine delivery of Cpf1 RNP together with ssDNA.

a) ssDNA does not increase the gene editing efficiency of the Cpf1 RNP with delivery using lipofectamine. ssDNA one hundred nucleotides in length, without sequence homology, was added to Cpf1 RNP to determine its effect on gene editing. Mean ± S.E, n=3. $p < 0.01$.

b) crRNAs extension and chemical modifications were delivered with lipofectamine together with ssDNA. The same transfection without ssDNA data are presented in Figure 5e, which shows generally higher GFP knock-out efficiency. Mean ± S.E, n=3.

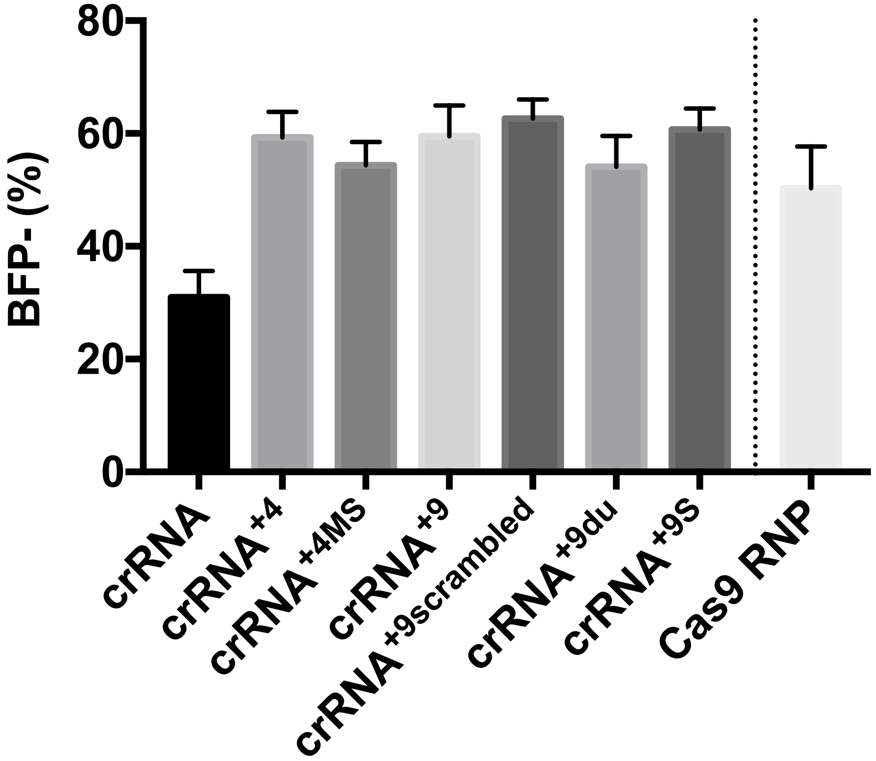

Supplementary Figure 2. Cpf1 RNP with 5'- extended crRNAs, which contain chemical modifications on the extension, have enhanced gene editing efficiency in BFP-HEK cells. crRNAs with 4 nt and 9 nt extensions were investigated that contained the following modifications. +MS refers to crRNA in which the first three nucleotides from the 5'-, have been modified with a 2'-OMe and phosphorothioate modifications. +9du refers to crRNA in which there is a 2'-deoxy modification on the 9th nucleotide from the 5' end. +9S refers to crRNA in which the first 9 nucleotides from 5'- have phosphorothioate modifications on the phosphate backbone and the 9th nucleotide also has a 2'-deoxy modification. BFP knock-out efficiency was measured via flow cytometry 7 days after electroporation. Mean ± S.E, n=3. All extended crRNAs with chemical modifications were statistically different to un-extended crRNA, and had a p value smaller than 0.05, determined by the student-t-test.

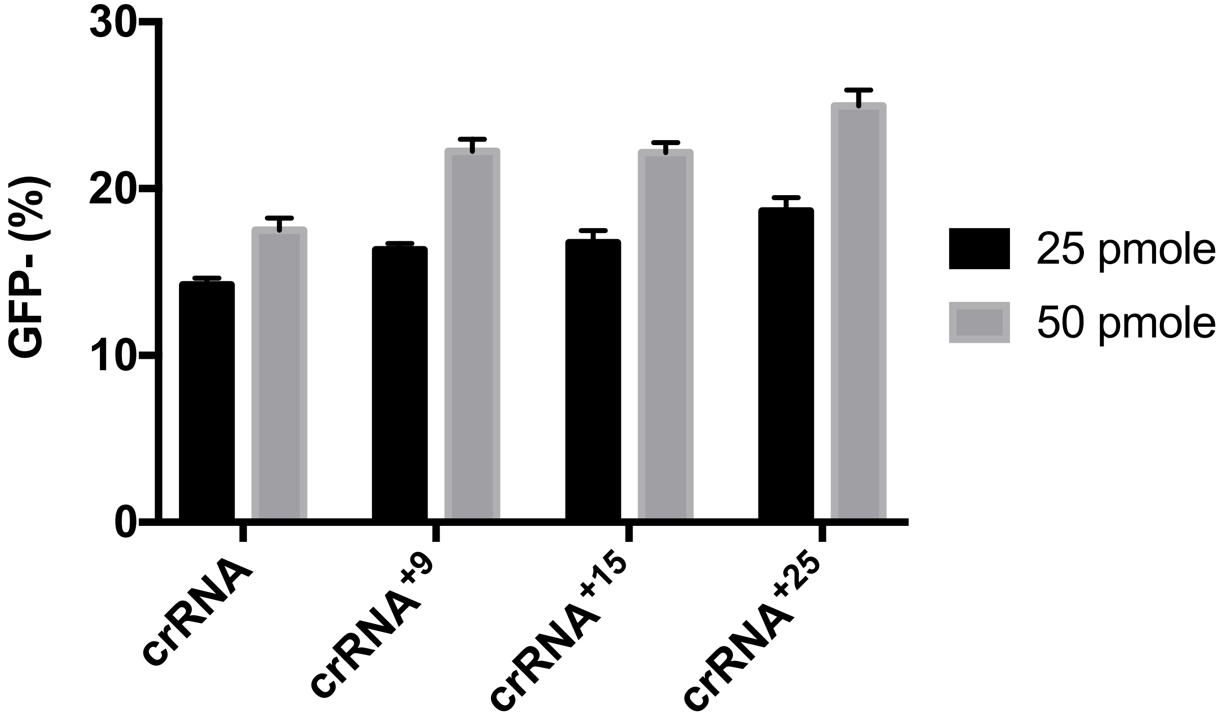

Supplementary Figure 3. Lipofectamine delivery of Cpf1 RNP with crRNA extensions. Cpf1 RNP (25 pmole or 50 pmole) with crRNA, cRNA+9, crRNA+15, or crRNA+25 were delivered to GFP-HEK cells using lipofectamine. The gene editing efficiency of the Cpf1 RNP increases with the length of crRNA. The gene editing efficiency of cells treated with 50 pmole of Cpf1 RNP is generally higher than cells treated with 25 pmole. Mean ± S.E, n=3. All extended crRNAs were statistically different to un-extended crRNA, and had a p value smaller than 0.05, determined by the student-t-test.

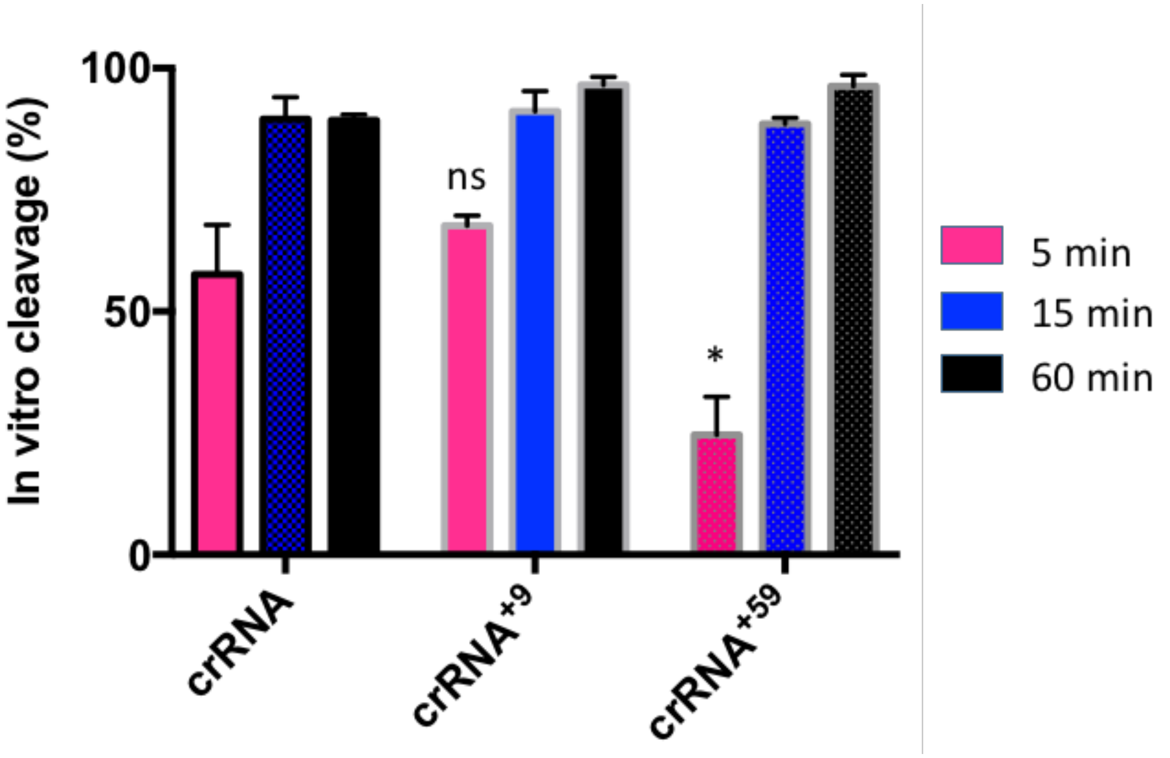

Supplementary Figure 4. Quantification of DNA cleavage by Cpf1 RNP complexed to various crRNAs. Cpf1 RNP with crRNA, crRNA+9, or crRNA+59 was incubated with the target DNA template for up to 60 min and analyzed by gel electrophoresis. Band intensity was quantified to calculate the percent cleavage. Mean ± S.E, n=3. *, $p < 0.05$. ns=non-significnat

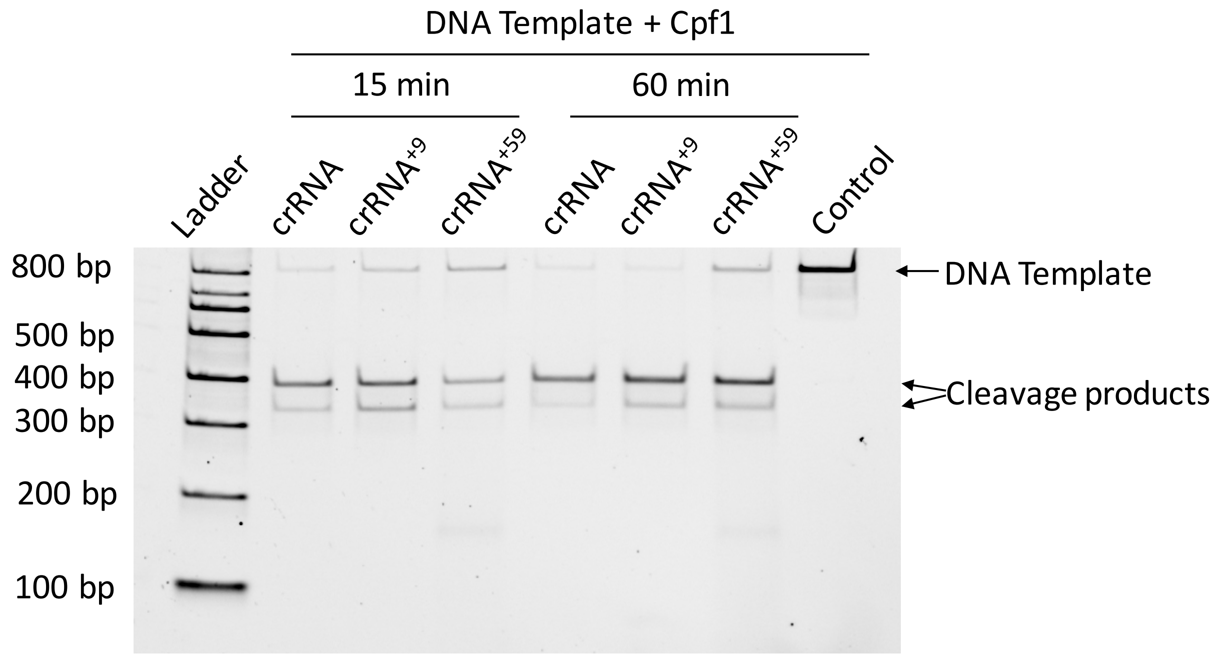

Supplementary Figure 5. Gel analysis of the in vitro DNA cleavage assay performed with Cpf1 RNP complexed to various crRNAs. Cpf1 RNP with crRNA, crRNA+9, or crRNA+59 was incubated with the DNA template for 15 min or 60 min. Gel electrophoresis using a PAGE gel separated the starting DNA template from the cleavage products.

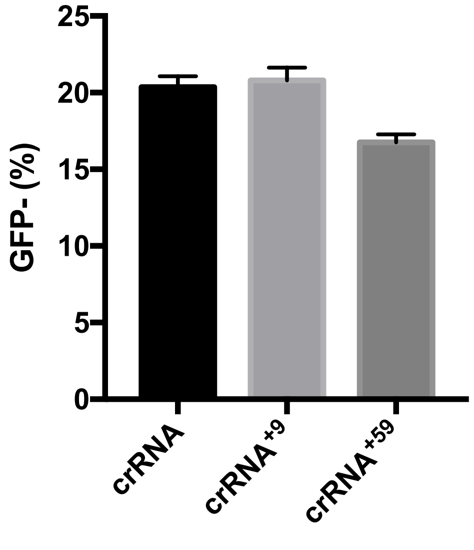

Supplementary Figure 6. Extended crRNAs show no enhancement of gene editing efficiency when Cpf1 plasmid was delivered instead of Cpf1 protein. Cpf1 plasmid was

delivered 24 hr in advance to crRNA delivery via electroporation in GFP-HEK cells. The GFP knock-out percentage was quantified by flow cytometry. $crRNA^{+59}$ shows a lower gene editing efficiency compared to crRNA ($p < 0.05$). Mean ± S.E, n=3.

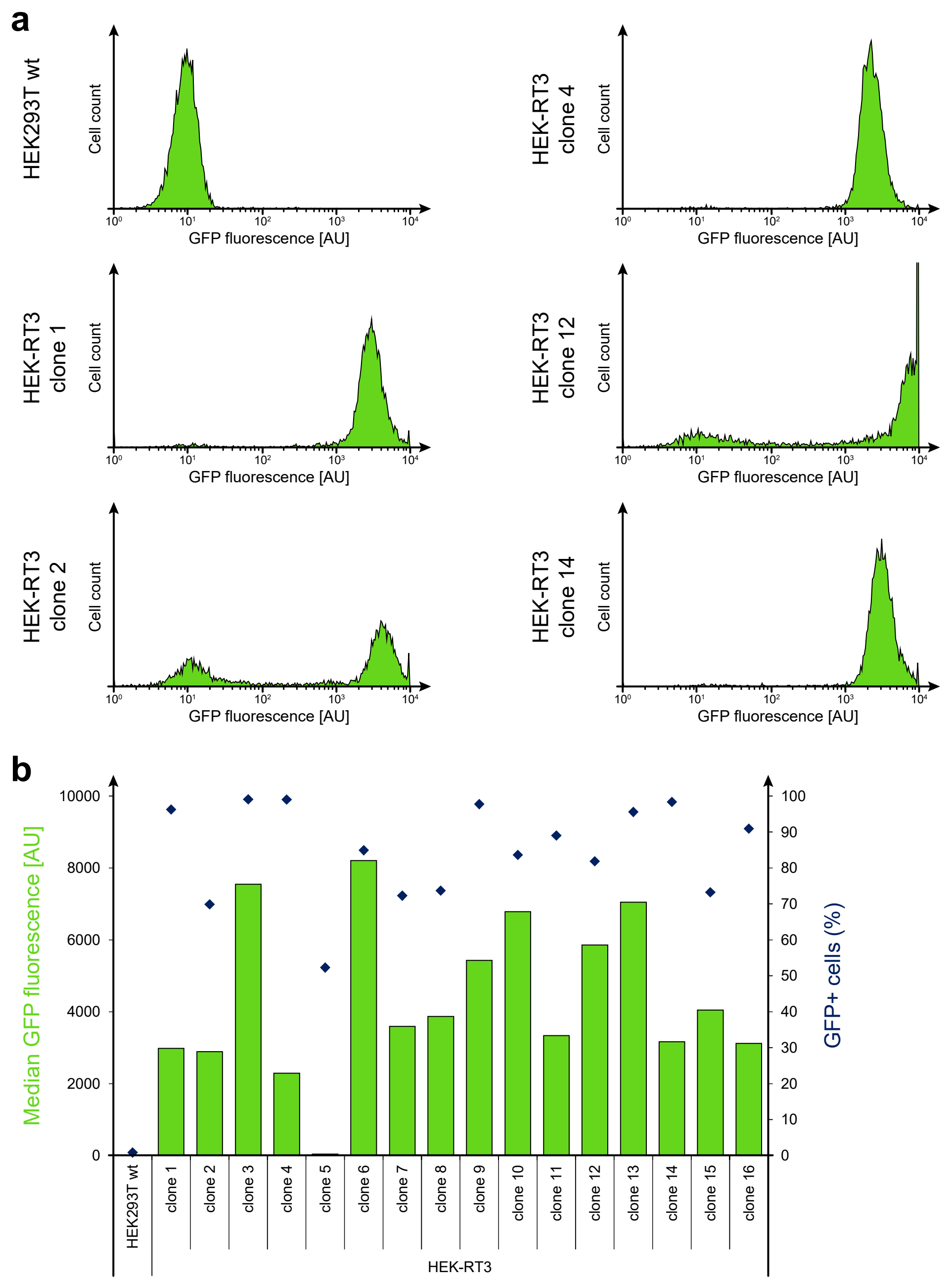

Supplementary Figure 7. GFP-HEK cell line generation. a) Flow cytometry analysis of GFP fluorescence in HEK293T (negative control, which has low fluorescence), and reporter cells infected with GFP containing retroviruses, the GFP expression was controlled via a doxycline inducable promoter. The reporter cells were generated by transducing HEK293T at low copy with the amphotropic pseudotyped retroviral vector RT3GEPIR, followed by isolation and characterization of monoclonal cell lines. Monoclonal GFP-HEK cell lines were treated with doxycline before performing flow cytometry. Representative clones are shown. The clone 4, also

referred to as “GFP-HEK”, was chosen for future experiments. b) Quantification of GFP fluorescence from flow cytometry data shown in a).

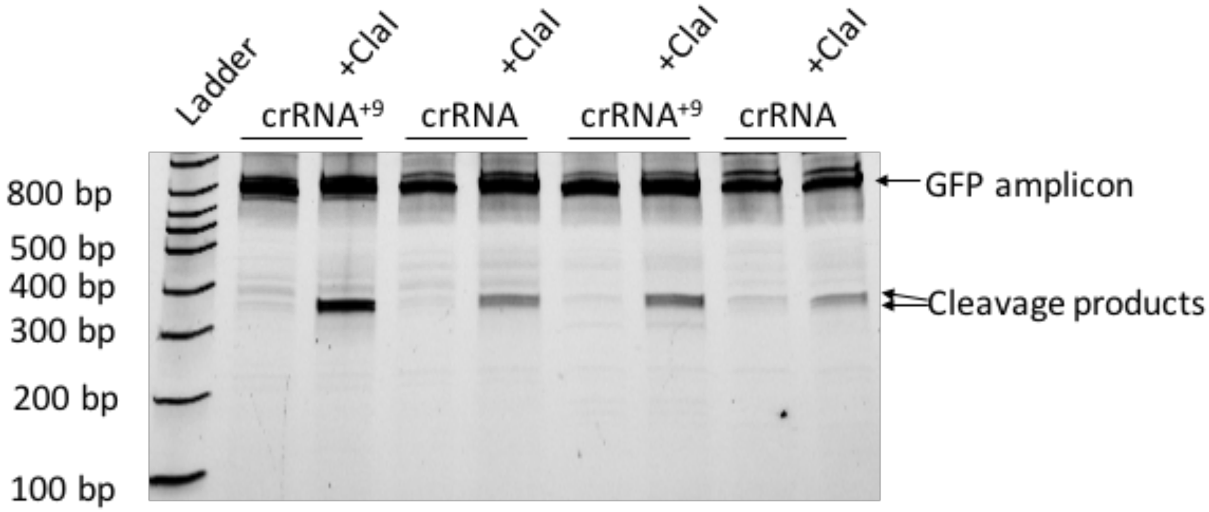

Supplementary Figure 8. A representative gel image of the restriction enzyme assay used to quantify HDR quantification in Figure 2b. Cpf1 RNP plus ssODN were delivered to GFP-HEK cells using electroporation. PCR amplification of the target gene and ClaI restriction enzyme cleavage followed to quantify HDR. Image Lab (Bio-rad) was used to quantify band intensity.

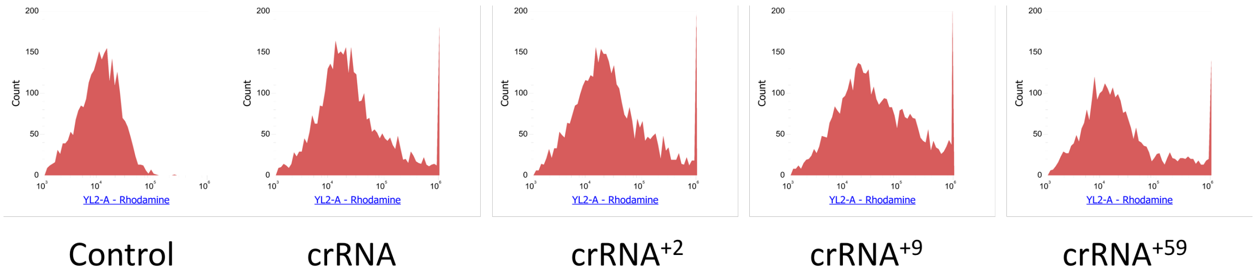

Supplementary Figure 9. Representative flow cytometry images of Figure 4b. RFP positive ai9 myoblasts were quantified by flow cytometry after electroporation of Cpf1 RNP with crRNAs of different lengths.

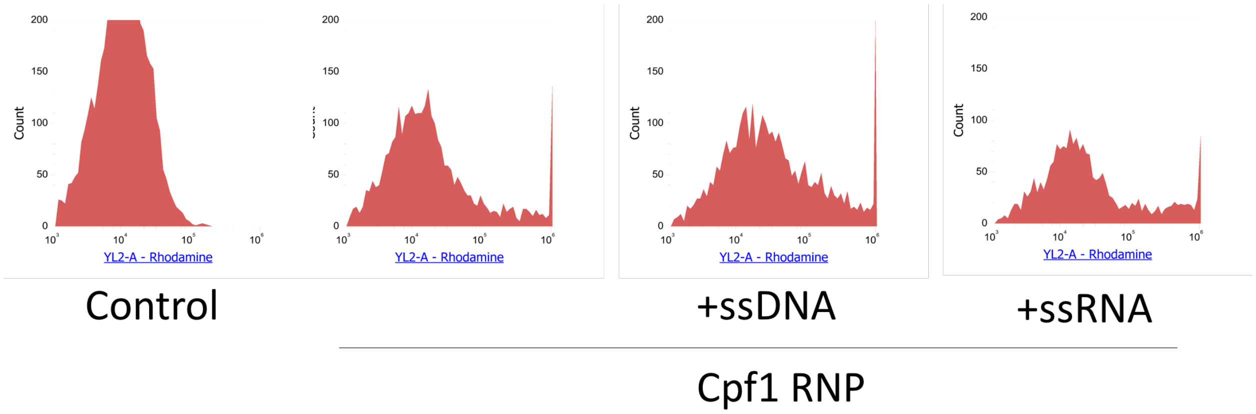

Supplementary Figure 10. Representative flow cytometry images of Figure 4c. RFP positive ai9 myoblasts were quantified by flow cytometry after electroporation of Cpf1 RNP with or without ssDNA/ssRNA.

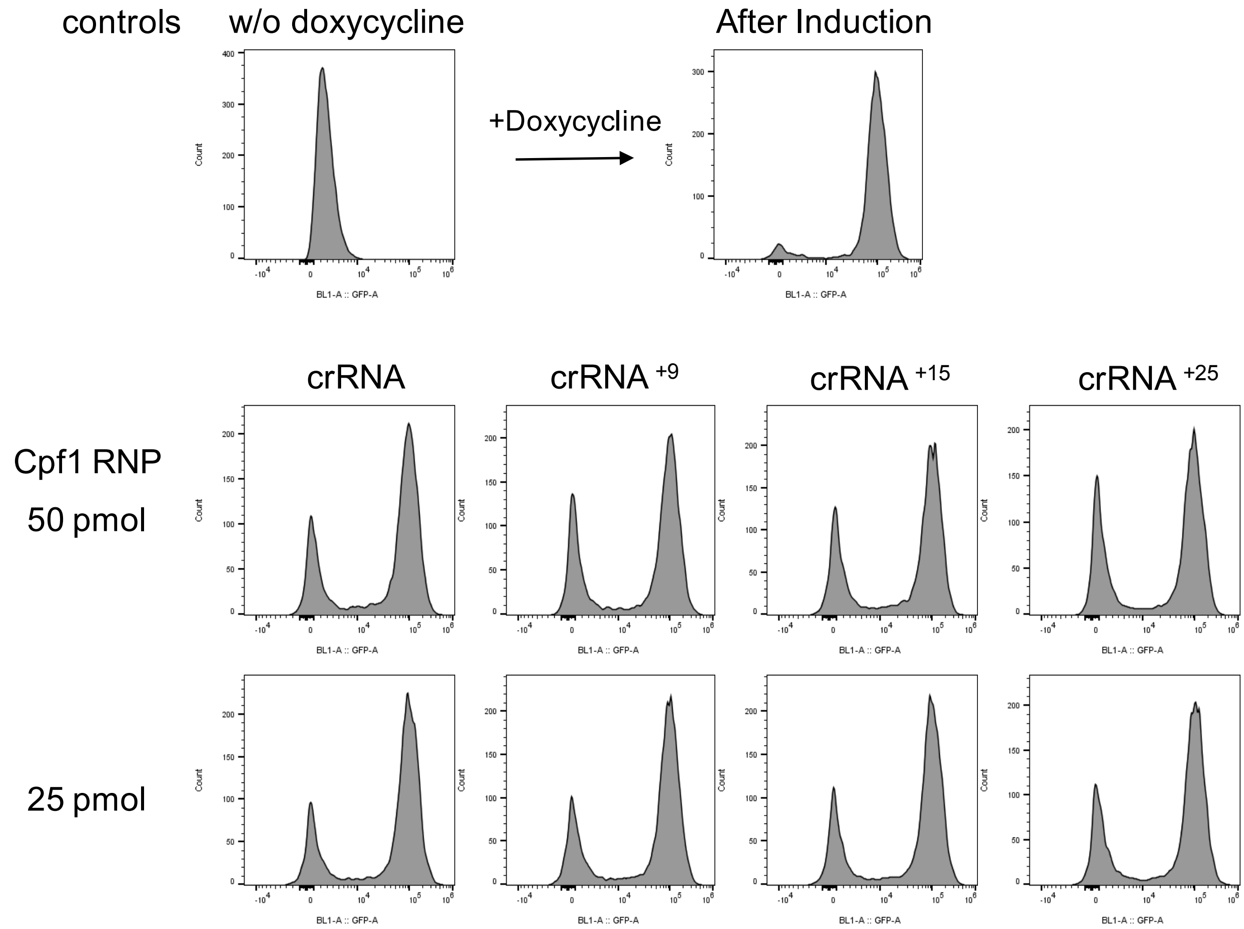

Supplementary Figure 11. Representative flow cytometry images of Supplementary Figure 3. Addition of doxycycline induces GFP expression in GFP-HEK cells. Transfection of Cpf1 RNP using lipofectamine induced knock-out of the GFP gene and a generated a population of GFP negative cells, even

after doxycycline induction. The degree of GFP negative cells were quantified and the background from the control was subtracted.

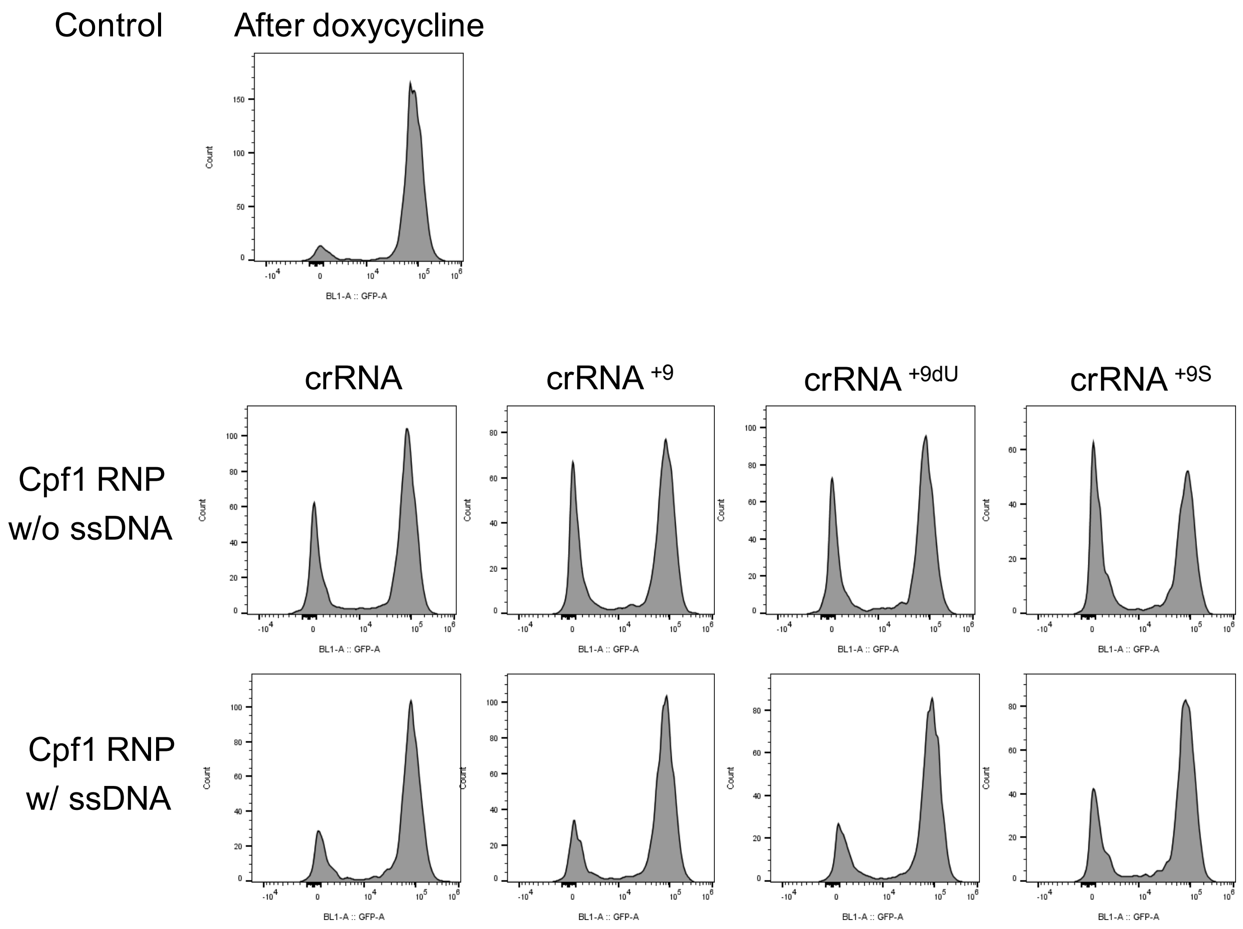

Supplementary Figure 12. Representative flow cytometry images of Supplementary Figure 5e and Supplementary Figure 2. Transfection of Cpf1 RNP with or without ssDNA using lipofectamine in GFP-HEK cells.

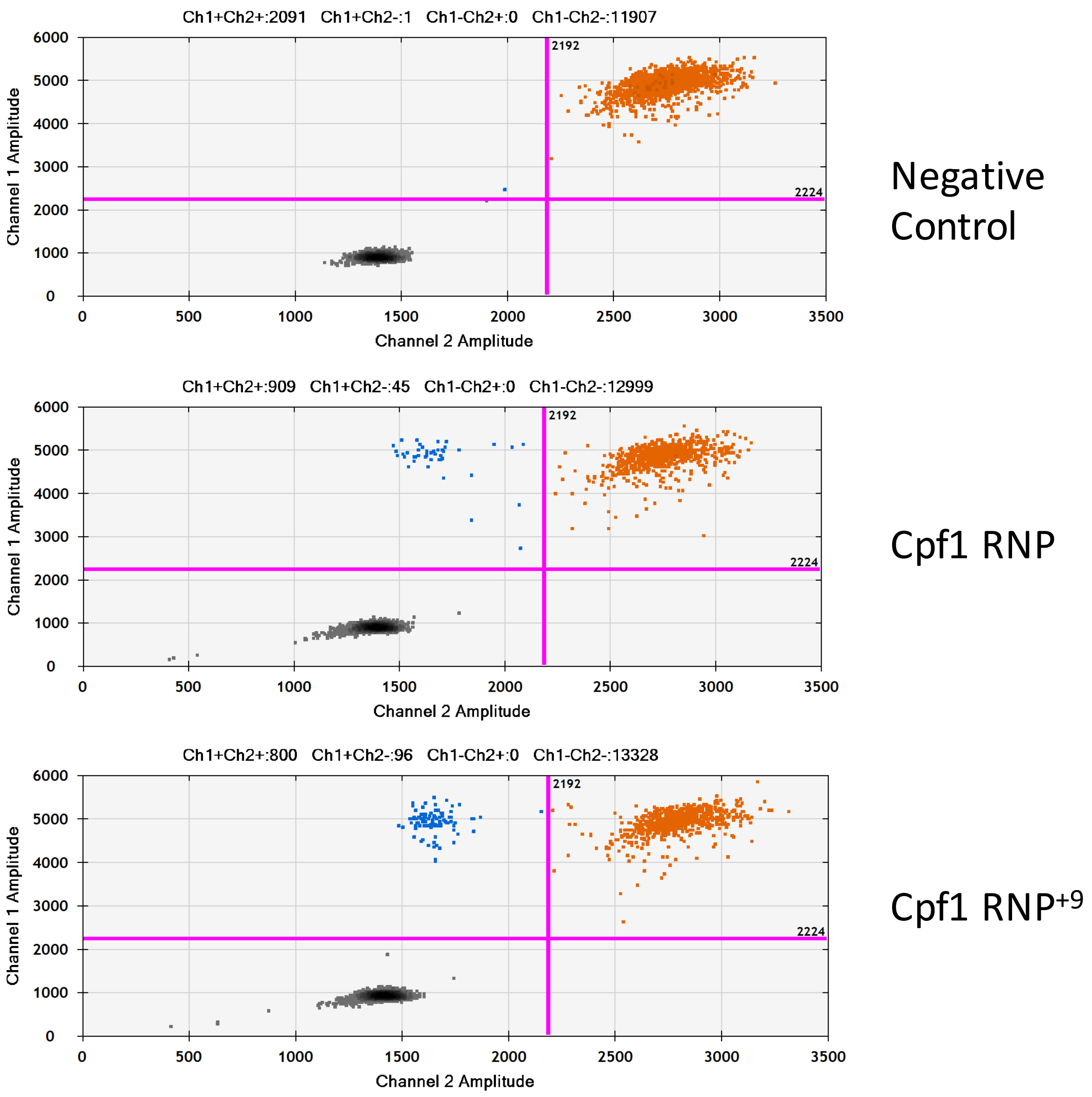

Supplementary Figure 13. Representative original ddPCR analysis images of Figure 4d.

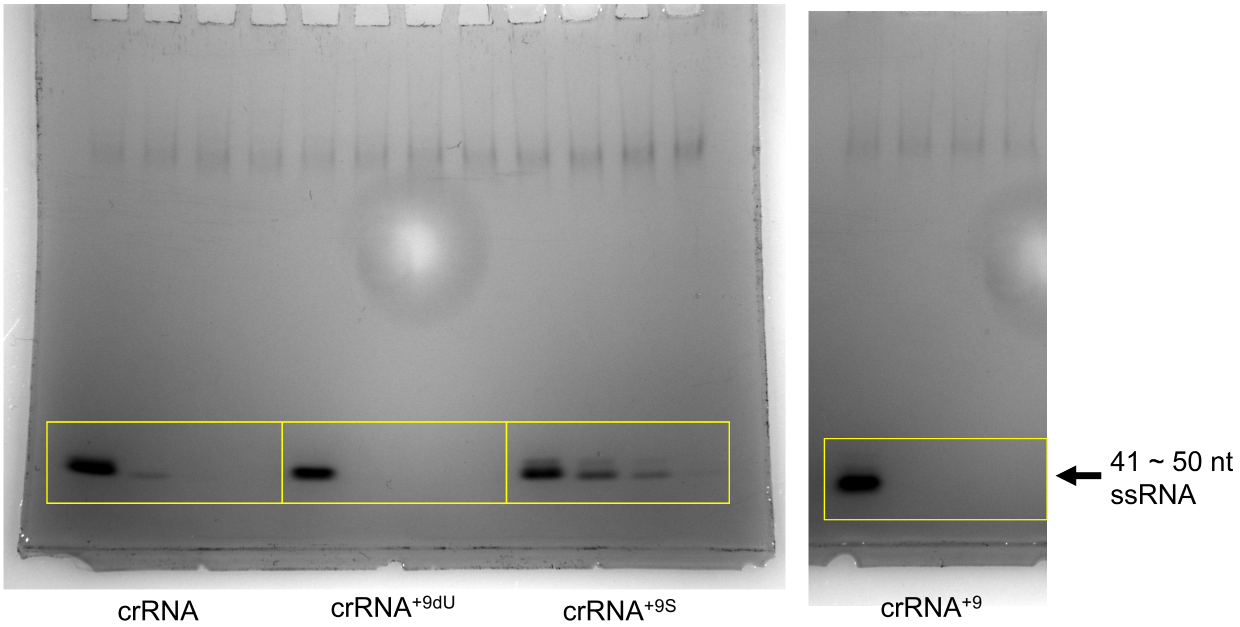

Supplementary Figure 14. Original gel image of the crRNAs with serum incubation in Figure 5c. Yellow boxes show cropped images for main figure 5c.
