# Supplementary material for: Extension of the crRNA enhances Cpf1 gene editing in vitro and in vivo
Source: Nat Commun. 2018 Aug 17;9:3313. doi: 10.1038/s41467-018-05641-3 (PMC6098076; doi:10.1038/s41467-018-05641-3)
Supplement: Supplementary file 3 — Description of Additional Supplementary Files [file 41467_2018_5641_MOESM3_ESM.pdf]

Description of Additional Supplementary Files

File Name: Supplementary Data 1
Description: Sequence information of nucleic acids.
